# Association of Changes in Vector Length with Changes in Left Ventricular Mass among Patients on Maintenance Hemodialysis: A Secondary Analysis of the Frequent Hemodialysis Network Daily Trial

**DOI:** 10.34067/KID.0000000000000443

**Published:** 2024-04-24

**Authors:** Enass Elsayed, Youssef M.K. Farag, Katherine Scovner Ravi, Glenn M. Chertow, Finnian R. Mc Causland

**Affiliations:** 1 Brigham and Women's Hospital, Boston, Massachusetts; 2 Harvard Medical School, Boston, Massachusetts; 3 Bayer US, LLC, Pittsburgh, Pennsylvania; 4 Department of Epidemiology, Johns Hopkins Bloomberg School of Public Health, Baltimore, Maryland; 5 Departments of Medicine, Epidemiology and Population Health, and Health Policy, Stanford University School of Medicine, Stanford, California

**Keywords:** cardiovascular, chronic hemodialysis, dialysis, heart failure, hemodialysis

## Abstract

**Key Points:**

- Bioimpedance has been proposed as an objective method to assess volume status among patients receiving maintenance hemodialysis.
- The Frequent Hemodialysis Network Daily Trial measured bioimpedance parameters of volume status (vector length) and cardiac magnetic resonance imaging at baseline and 12 months.
- We observed that changes in vector length were inversely associated with changes in left ventricular mass and volume over a 12-month period.

**Background:**

Hypervolemia is thought to be a major contributor to higher left ventricular mass (LVM), a potent predictor for cardiovascular mortality among patients on maintenance hemodialysis. We hypothesized that a decrease in vector length (a bioimpedance proxy of hypervolemia) would be associated with an increase in LVM.

**Methods:**

Using data from the Frequent Hemodialysis Network Daily Trial (*n*=160), we used linear regression to assess the association of changes in vector length from baseline to month 12 with changes in magnetic resonance imaging measures of LVM and other cardiac parameters. We adjusted models for the randomized group, baseline vector length, age, sex, race, body mass index, vascular access, dialysis vintage, history of hypertension, heart failure, and diabetes, residual kidney function, predialysis systolic BP, ultrafiltration rate, serum-dialysate sodium gradient, hemoglobin, phosphate, angiotensin-converting enzyme inhibitor or angiotensin receptor blocker use, erythropoietin dose, and equilibrated Kt/V.

**Results:**

The mean age of the patients was 50±13 years; 35% were female. In the fully adjusted models, a decline in vector length (per 50 Ω/m; *i.e*., increase in volume) was associated with a 6.8 g (95% confidence interval [CI], −0.1 to 13.7) and 2.6 g/m^2^ (95% CI, −1.2 to 6.3) increase in LVM and LVM index, respectively, and an increase of 15.0 ml (95% CI, 7.5 to 22.4), 7.3 ml (95% CI, 3.0 to 12.7), 7.8 ml (95% CI, 3.0 to 12.7), and −0.9% (95% CI, −3.1 to 1.3) in left ventricular end-diastolic volume, left ventricular end-systolic volume, left ventricular stroke volume, and left ventricular ejection fraction, respectively. The lowest tertile of change in vector length (*i.e*., greater increase in volume) was associated with greater increases in left ventricular end-diastolic volume and left ventricular stroke volume, versus the highest tertile. There was no evidence of heterogeneity by randomized group.

**Conclusions:**

Change in vector length, a bioimpedance-derived proxy of volume status, was inversely associated with indices of LVM and volume measured by cardiac magnetic resonance imaging in patients randomized to conventional or frequent hemodialysis over 12 months.

## Visual Abstract

**Figure:**
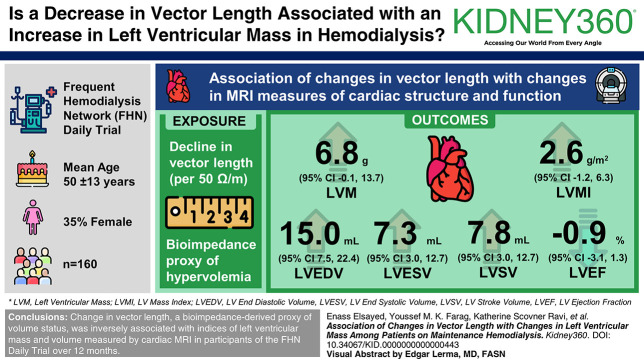

## Introduction

Cardiovascular disease remains the leading cause of mortality among patients with ESKD receiving maintenance hemodialysis, accounting for around 40% of deaths.^1^

Cardiac structural abnormalities tend to accumulate with the progression of CKD such that left ventricular hypertrophy (LVH) is estimated to affect 75% of patients initiating maintenance hemodialysis therapy.^2–4^ LVH and higher left ventricular mass (LVM) are potent predictors of cardiovascular mortality among patients receiving maintenance hemodialysis^5,6^ while observational data suggest that regression of LVH is associated with lower mortality.^3^ Similarly, higher left ventricular (LV) volume is a powerful independent predictor of death in patients with structural heart disease.^7,8^

Hypervolemia, estimated to affect 56%–73% of patients receiving maintenance hemodialysis,^9^ is thought to contribute to changes in LV structure and function.^10^ Clinical assessment of volume status has inherent limitations^11^ while data regarding the association of more objective measures of volume status (and changes over time) with sensitive measurements of cardiac indices (cardiac magnetic resonance imaging [MRI]) remain sparse.

Therefore, using detailed data from the Frequent Hemodialysis Network (FHN) Daily Trial, we tested the hypothesis that changes in vector length, a bioimpedance-derived proxy of volume status, are associated with changes in LV structure and function assessed by cardiac MRI. Furthermore, we tested whether the associations differed according to the randomized treatment arm (6/wk versus 3/wk hemodialysis).

## Methods

### Study Design and Population

The FHN Daily Trial was a multicenter, randomized, parallel-group trial comparing frequent (6/wk) with conventional (3/wk) in-center hemodialysis, conducted in the United States and Canada.

Patients undergoing maintenance hemodialysis were considered for enrollment if they were at least 13 years old, attained a mean equilibrated Kt/V urea value >1.0 during their past two baseline hemodialysis sessions, and had a body weight exceeding 30 kg. Notable factors that led to exclusion from the trial were inadequate treatment adherence, inability to use heparin, residual urea clearance >3 ml/min per 35 L, undergoing hemodialysis for fewer than three months, and being unable to undergo cardiac MRI.

The study design and protocol,^12,13^ primary results,^14^ and results of several secondary analyses of the FHN Daily Trial have been published.^15–17^ The protocol was approved by institutional review boards at each participating center, and written informed consent was obtained from all study participants. We obtained data for the present analyses from the National Institute of Diabetes and Digestive and Kidney Diseases data repository.

Two coprimary composite outcomes were assessed in the original trial: (*1*) death or change (from baseline to 12 months) in LVM and (*2*) death or change (from baseline to 12 months) in the physical health composite score of the research and development 36-item health survey.

### Exposure Variables

We considered changes in vector length from baseline to the end of the follow-up period (month 12–month 0) as the primary exposure of interest. These measurements were obtained with single-frequency (50 Hz) bioelectrical impedance analysis, using the Hydra 4200 Bioimpedance Analyzer (San Diego, CA) just before a mid-week hemodialysis session with the patient in a recumbent position for those with at least one intact leg and arm; however, a minority of bioelectrical impedance analysis assessments were performed on other days or after hemodialysis. We calculated vector length indexed to height in meters (Z/H) from the raw measurements of resistance (R) and reactance (Xc), where R represents the opposition to the flow of an alternating current through ionic solutions and Xc is the capacitance produced by interfaces across tissues (*e.g*., cell membranes), according to the following formula: |Z/H|=√[(R/H)^2^+(Xc/H)^2^].^18,19^

We considered change in vector length in both a continuous and categorical (tertiles) fashion. A positive value for the change in vector length reflects a decrease in soft-tissue hydration from baseline to month 12; conversely, a negative value reflects an increase in soft-tissue hydration from baseline to month 12. The highest tertile of change was chosen as the reference; the lowest tertile of change, therefore, reflects an increase in soft-tissue hydration from baseline to month 12.

### Outcomes

The primary outcome of interest was change in LVM, as assessed by cardiac MRI from baseline to 12 months after randomization. Secondary outcomes included changes from baseline to 12 months in LVM index (LVMI), LV end-diastolic volume (LVEDV), LV end-systolic volume (LVESV), LV stroke volume (LVSV), and LV ejection fraction (LVEF).

Cardiac MRI was performed using the 1.5-T MRI systems (minimum gradient performance: peak strength ≥12 mT/m, slew rate ≥40 mTm/s) with dedicated surface coils. Standardized protocols were used across centers with central and blinded review of acquired images.^14,15^ Myocardial volume (excluding papillary muscles) was measured on end-diastolic frames using validated software. The derived volume was multiplied by the specific density of the myocardium (1.05 g/cm^3^) to calculate LVM^15^ and indexed to body surface area using the formula of DuBois and DuBois.^20^

### Statistical Analyses

We examined continuous variables graphically and reported values as means (±SDs) for normally distributed data or medians (25th–75th percentiles) for non-normally distributed data. We examined categorical variables by frequency distribution and reported values as proportions. We compared baseline characteristics across tertiles of the change in vector length using tests for trend on the basis of linear regression, chi-square trend test, and the Cuzick nonparametric trend test, as appropriate for data distribution.

We assessed the association of the change in vector length with the change in LV indices from baseline to month 12 using unadjusted and adjusted linear regression models. The multivariable model was adjusted for randomized treatment assignment, predialysis systolic BP, baseline vector length, age, sex, self-reported race, Quételet (body mass) index (body mass index), vascular access type (arteriovenous fistula, graft, or tunneled catheter), dialysis vintage (<2, 2–5, >5 years), history of hypertension, heart failure, diabetes, residual kidney function (0, ≤1, >1–3, >3 ml/min), hemoglobin, serum phosphorus, ultrafiltration rate, angiotensin-converting enzyme inhibitor or angiotensin receptor blocker use, log-transformed erythropoietin dose, and equilibrated Kt/V. For each separate cardiac MRI parameter, the corresponding baseline measurement was included in the multivariable model. A further model was considered that was additionally adjusted for serum sodium to dialysate gradient—this was considered as an exploratory sensitivity analysis because data were missing from 27% of sessions. Other covariates had complete data, apart from one missing hemoglobin value. Because all models considered the change from baseline to month 12 for the exposure and outcome and was adjusted for baseline covariates, there was no violation of the assumption of independence of observations, allowing the use of linear regression models. We assessed nonlinearity using restricted cubic splines. We assessed effect modification according to the randomized treatment arm by inclusion of cross-product terms in the adjusted model.

We conducted all analyses at an alpha level of 0.05, without correction for multiple hypothesis testing, using Stata MP (version 16.0, Stata Corp., College Station, TX).

## Results

### Baseline Characteristics

**Figure 1:**
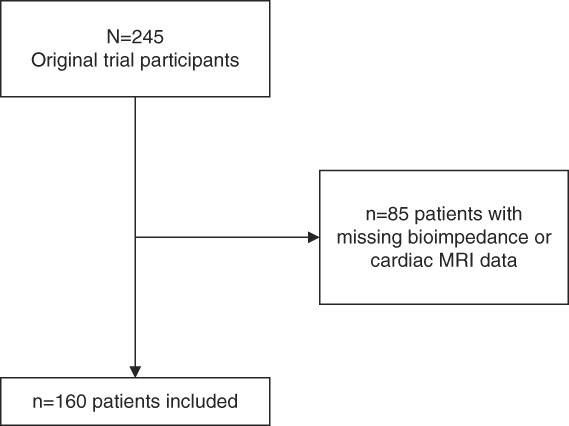
**CONSORT diagram.** MRI, magnetic resonance imaging.

A total of 160 (65%) of the original 245 patients had cardiac MRI and bioimpedance data at baseline and 12 months and were included in the present analyses (Figure 1). A comparison of the baseline characteristics of included versus excluded patients is presented in Supplemental Table 1.

Of the patients included in the present analyses, at baseline, the mean age was 50±13 years, 35% were women, and 39% had diabetes mellitus. At baseline, those in the lowest tertile of change in vector length (*i.e*., largest increase in volume from baseline to 12 months) were more likely to be older, have higher hemoglobin, be randomized to 3/wk hemodialysis, have lower ultrafiltration rates, and were less likely to have hypertension (Table 1). There were no major differences in the cardiac MRI parameters at baseline or 12 months across the tertiles of change in vector length (Table 2).

**Table 1:** Baseline characteristics according to categories of change in vector length

Characteristic	Categories of Change in Vector Length (Ω/m) from Baseline to Month 12			*P* Trend
	Tertile 1 -41±26 Ω/m (n=54)	Tertile 2 4±10 Ω/m (*n*=53)	Tertile 3 54±27 Ω/m (*n*=53)	
Baseline vector length, Ω/m	315±65	276±63	261±55	<0.001
Age, yr	51±14	53±14	45±9	0.03
**Sex, *n* (%)**				0.59
Female	21 (39)	17 (32)	18 (34)	
**Race or ethnic group, *n* (%)**				0.89
Native American, Aboriginal Canadian, Alaskan Native, or First Nation	1 (1.9)	3 (5.7)	2 (3.8)	
Asian	4 (7.4)	2 (3.8)	7 (13.2)	
Native Hawaiian or another Pacific Islander	0 (0.0)	1 (1.9)	2 (3.8)	
Black	28 (51.9)	26 (49.1)	18 (34.0)	
White	14 (25.9)	18 (34.0)	17 (32.1)	
Multiracial, unknown or not reported	7 (13.0)	3 (5.7)	7 (13.2)	
BMI, kg/m^2^	27.3±6.9	27.7±6.6	26.5±6.2	0.54
**Dialysis access, *n* (%)**				0.02
Graft	15 (28.3)	5 (9.4)	6 (11.5)	
Fistula	31 (58.5)	36 (67.9)	34 (65.4)	
Catheter	7 (13.2)	12 (22.6)	12 (23.1)	
**Duration of ESKD, yr, *n* (%)**				0.86
<2	11 (20.4)	23 (43.4)	13 (24.5)	
2–5	23 (42.6)	10 (18.9)	17 (32.1)	
>5	20 (37.0)	20 (37.7)	23 (43.4)	
**Coexisting medical conditions, *n* (%)**				
Hypertension	47 (87.0)	48 (90.6)	52 (98.1)	0.04
Heart failure	11 (20)	9 (17)	11 (21)	0.96
Diabetes mellitus	22 (40.7)	20 (37.7)	20 (37.7)	0.75
**KrU, *n* (%)**				0.54
Anuria	35 (64.8)	30 (56.6)	39 (73.6)	
>0–1 ml/min	11 (20.4)	7 (13.2)	5 (9.4)	
>1–3 ml/min	8 (14.8)	15 (28.3)	8 (15.1)	
>3 ml/min	0 (0.0)	1 (1.9)	1 (1.9)	
Predialysis systolic BP, mm Hg	146±17	150±20	147±18	0.79
**Predialysis laboratory results, mg/dl**				
Hemoglobin	12.3±1.3	12.0±1.2	11.8±1.2	0.05
Serum phosphate	5.6±1.6	5.6±1.8	6.1±1.4	0.11
Kt/V equilibrated	1.43±0.27	1.40±0.28	1.44±0.24	0.76
Ultrafiltration rate, ml/kg per hour	11.4±3.4	11.9±4.0	12.9±4.5	0.05
Sodium gradient, mmol/L	−2 (−4 to −0)	−1 (−4 to 1)	−2 (−6 to 1)	0.88
ACEi or ARB use, *n* (%)	25 (46.3)	19 (35.8)	34 (64.2)	0.07
Erythropoietin dose, Units	9862 (4500–21,000)	6400 (2700–18,750)	8500 (3275–12,125)	0.26
Randomized to 6/wk hemodialysis, *n* (%)	21 (38.9)	27 (50.9)	38 (71.7)	<0.001

The results are presented as mean±SD or median (25th–75th percentiles) for continuous variables. ACEi, angiotensin-converting enzyme inhibitor; ARB, angiotensin receptor blocker; BMI, body mass index; KrU, residual renal urea clearance.

**Table 2:** Baseline and 12-month cardiac magnetic resonance imaging parameters according to categories of change in vector length

Characteristic	Categories of Change in Vector Length (Ω/m) from Baseline to Month 12			*P* Value
	Tertile 1 −41±26 Ω/m (*n*=54)	Tertile 2 4±10 Ω/m (*n*=53)	Tertile 3 54±27 Ω/m (*n*=53)	
**LV mass, g**				
Baseline	137±46	143±57	149±64	0.54
Month 12	132±47	133±57	129±46	0.91
**LVMI, g/m** ^**2**^				
Baseline	71±23	73±29	80±32	0.28
Month 12	68±24	68±28	70±25	0.92
**LVEDV, ml**				
Baseline	173±53	171±60	184±60	0.42
Month 12	171±42	164±58	155±42	0.22
**LVESV, ml**				
Baseline	78±40	72±32	81±38	0.43
Month 12	75±32	71±35	64±26	0.19
**LVSV, ml**				
Baseline	94±26	99±35	103±36	0.37
Month 12	96±26	93±31	91±23	0.60
**LVEF, %**				
Baseline	56±11	59±9	57±11	0.40
Month 12	57±11	58±10	60±8	0.44

LV, left ventricular; LVEDV, left ventricular end-diastolic volume; LVEF, left ventricular ejection fraction; LVESV, left ventricular end-systolic volume; LVMI, left ventricular mass index; LVSV, left ventricular stroke volume.

### Association of Change in Vector Length with Changes in LVM and LVMI

The median change in vector length from baseline to month 12 was +5 (−20, +34) Ω/m. In unadjusted analysis, a more pronounced decline in vector length from baseline to month 12 (per 50 Ω/m; *i.e*., generally corresponding to an increase in volume) was associated with an increase in LVM (10.1; 95% confidence interval [CI], 4.6 to 15.6 g) and LVMI (5.1; 95% CI, 2.1 to 8.0 g/m^2^). In fully adjusted models, these associations were attenuated: LVM (6.8; 95% CI, −0.1 to 13.7 g) and LVMI (2.6; 95% CI, −1.2 to 6.3 g/m^2^) per 50 Ω/m decline in vector length from baseline to 12 months (Table 3). In additional analyses adjusting for sodium gradient, effect estimates were accentuated: LVM (13.5; 95% CI, 4.2 to 22.7 g) and LVMI (5.5; 95% CI, 0.6 to 10.5 g/m^2^) per 50 Ω/m decline in vector length from baseline to 12 months (Supplemental Table 2).

**Table 3:** Association of change in vector length with change in cardiac magnetic resonance imaging parameters

Outcomes	Model	Change in Outcome from Baseline to Month 12 According to Change in Vector Length (95% CI)					*P* Trend
		Per 50 Ω/m Decrease in Vector Length	*P* Value	Tertile 1	Tertile 2	Tertile 3	
LVM, g	Unadjusted	10.1 (4.6 to 15.6)	<0.001	15.0 (2.7 to 27.3)	9.3 (−3.1 to 21.6)	Ref	0.02
	Adjusted	6.8 (−0.1 to 13.7)	0.05	6.5 (−8.2 to 21.1)	7.0 (−6.3 to 20.3)	Ref	0.37
LVMI, g/m^2^	Unadjusted	5.1 (2.1 to 8.0)	0.001	6.7 (0.2 to 13.3)	4.3 (−2.3 to 10.9)	Ref	0.04
	Adjusted	2.6 (−1.2 to 6.3)	0.17	0.5 (−7.4 to 8.4)	2.2 (−5.1 to 9.4)	Ref	0.89
LVEDV, ml	Unadjusted	16.3 (9.2 to 23.4)	<0.001	27.5 (11.6 to 43.4)	23.3 (7.4 to 39.3)	Ref	0.001
	Adjusted	15.0 (7.5 to 22.4)	<0.001	25.4 (9.3 to 41.6)	15.6 (0.9 to 30.3)	Ref	0.002
LVESV, ml	Unadjusted	9.4 (4.7 to 14.0)	<0.001	13.4 (2.9 to 23.9)	15.8 (5.3 to 26.3)	Ref	0.01
	Adjusted	7.3 (1.9 to 12.7)	0.01	9.2 (−2.5 to 20.8)	9.5 (−1.0 to 20.0)	Ref	0.11
LVSV, ml	Unadjusted	6.9 (2.2 to 11.6)	0.004	14.1 (3.7 to 24.4)	7.5 (−2.9 to 17.9)	Ref	0.01
	Adjusted	7.8 (3.0 to 12.7)	0.002	15.8 (5.5 to 26.1)	6.7 (−2.7 to 16.0)	Ref	0.003
LVEF, %	Unadjusted	−1.7 (−3.4 to 0.1)	0.06	−1.8 (−5.6 to 2.0)	−3.1 (−6.9 to 0.7)	Ref	0.36
	Adjusted	−0.9 (−3.1 to 1.3)	0.41	−0.3 (−4.9 to 4.3)	−0.9 (−5.1 to 3.2)	Ref	0.89

The multivariable model was adjusted for baseline vector length, baseline outcome, randomized treatment assignment, age, sex, race, body mass index, access type, vintage (<2, 2–5, >5 years), predialysis systolic BP, hypertension, heart failure, diabetes, residual urea clearance (0, ≤1, >1–3, >3 ml/min), hemoglobin, phosphate, ultrafiltration rate, angiotensin-converting enzyme inhibitor or angiotensin receptor blocker use, log-transformed erythropoietin dose, and equilibrated Kt/V. CI, confidence interval; LVEDV, left ventricular end-diastolic volume; LVEF, left ventricular ejection fraction; LVESV, left ventricular end-systolic volume; LVM, left ventricular mass; LVMI, left ventricular mass index; LVSV, left ventricular stroke volume.

In the unadjusted categorical analyses, an inverse association was observed between change in vector length with change in LVM and LVMI from baseline to month 12 (Table 3). However, these associations only approached statistical significance for LVM in the adjusted models that included the subset of patients with non-missing data on the serum-to-dialysate sodium gradient (Supplemental Table 2).

### Association of Change in Vector Length with the Changes in LVEDV, LVESV, LVSV, and LVEF

In adjusted analyses, a more pronounced decline in vector length from baseline to month 12 (per 50 Ω/m; *i.e*., increase in volume) was associated with an increase from baseline to month 12 in LVEDV, LVESV, and LVSV, but not with changes in LVEF (Table 3). There was no evidence for a nonlinear association of change in vector length with change in LVM, LVMI, LVEDV, LVESV, LVSV, or LVEF (*P* for nonlinearity = 0.18, 0.32, 0.19, 0.10, 0.85, and 0.73, respectively; Supplemental Figure 1). Similar patterns were noted in models where change in vector length was considered as a categorical variable and in models that included the subset of patients with non-missing data on the serum-to-dialysate sodium gradient (Table 3 and Supplemental Table 2).

### Assessment for Differential Associations according to Randomized Treatment Arm

In the fully adjusted model, there was no evidence for effect modification of the association of changes in vector length with LVM, LVMI, LVEDV, LVESV, LVSV, or LVEF (*P* interaction = 0.67, 0.39, 0.74, 0.63, 0.23, and 0.22, respectively). Subgroup analyses according to the randomized treatment arm are presented in Supplemental Table 3.

## Discussion

In this *post hoc* analysis of the FHN Daily Trial, we observed that decreases in vector length from baseline to 12 months, a proxy for volume expansion, were associated with increases in cardiac MRI determinations of LVM and indices of LV volume over the same period. These associations were not modified by the randomized treatment assignment of 6/wk versus 3/wk hemodialysis.

Cardiac structural abnormalities are common among patients initiating maintenance hemodialysis, with prior echocardiographic studies estimating that around 75% of patients meet the criteria for LVH, 36% had evidence for LV dilation, and 15% had evidence of systolic dysfunction.^2^ Somewhat similar estimates of LVH prevalence of 64% have been documented using cardiac MRI,^21^ which is widely recognized to provide more accurate assessments of cardiac dimensions than echocardiography in this patient population.^22^ Importantly, LVH and higher LVM are potent predictors of mortality among patients receiving maintenance hemodialysis.^23,24^ As such, changes in LVM have sometimes been considered as potentially modifiable surrogate end points for clinical trials (including as a coprimary end point for the FHN Daily trial).^14^

Although there are many potential etiologies for the development of higher LVM and other cardiac structural changes among patients receiving hemodialysis,^15^ unremitting hypervolemia is thought to play a major role,^10,25^ and has itself been independently associated with hospitalization and cardiovascular-related mortality.^26^ A prior study of prevalent patients (maintenance hemodialysis for >3 months; *n*=246) reported that higher end-diastolic volume, prehemodialysis systolic BP, and calcium–phosphate product were independent predictors of LVH and higher LVMI.^21^ The observation that higher end-diastolic volume was the strongest predictor of LVH and LVMI is consistent with the contention that sustained hypervolemia results in maladaptive responses of the LV in the setting of kidney failure.

To date, few studies have examined the association of bioimpedance proxies of volume status with echocardiographic or cardiac MRI assessments of cardiac structure and function among patients with kidney failure. One modest-sized cross-sectional study of Italian patients on maintenance hemodialysis (*n*=110) reported that higher extracellular water (derived from bioimpedance measurements) was independently and directly correlated with LVMI, assessed by echocardiography.^27^ Other cross-sectional observational studies in patients with stage 5 CKD, but not yet on hemodialysis, reported similar findings.^28,29^ Our present findings, therefore, expand the knowledge base in this regard, supporting the notion that changes in vector length over a 12-month period are significantly associated with changes in cardiac MRI parameters of LV mass and volume.

The primary results of the FHN Daily Trial demonstrated that frequent hemodialysis (compared with conventional, thrice-weekly hemodialysis) resulted in a relative reduction in LVM.^14^ A *post hoc* analysis of FHN also reported that randomization to 6/wk hemodialysis resulted in more profound reduction in LVEDV, compared with 3/wk hemodialysis.^30^ Furthermore, they observed that these effects differed by residual urine volume and were most apparent among those with urine volume ≤100 versus >100 ml/d (−14.2 versus −3.25 ml, respectively; *P* interaction = 0.02). One of the hypotheses put forward to explain these observations was related to improved overall volume status, which was also suggested from a smaller randomized cross-over trial of daily versus thrice-weekly hemodialysis, where concomitant reductions in bioimpedance metrics of extracellular volume and LVM were noted.^31^ Our present results support this hypothesis and, as evidenced by the lack of differential associations according to the randomized treatment arm, additionally suggest that the modality by which optimization of volume is achieved may not be paramount. In this respect, given the association of higher dialysate sodium with interdialytic weight gain on one hand and a lower risk of intradialytic hypotension on the other hand,^32^ it is notable that the effect estimates were more pronounced in models that adjusted for the serum-to-dialysate sodium gradient. However, a prior randomized controlled trial of conventional versus lower dialysate sodium (140 versus 135 mmol/L) reported no differences in cardiac MRI-assessed LVMI over 12 months, highlighting the need for further research in this area.^33^

The strengths of our study include the availability of repeated measures of bioimpedance and cardiac MRI performed in the setting of a randomized controlled trial. Furthermore, we were able to perform multivariable-adjusted models to account for potential confounders, including ultrafiltration rates and the serum-to-dialysate sodium gradient. However, there were several limitations to consider. These include the potential for residual confounding and risk of false-positive results from multiple testing in this *post hoc* observational analysis, lack of detailed information on dietary sodium intake and sodium balance, and lack of data on natriuretic peptides. Further limitations relate to the generalizability of our findings to patients beyond those included in the FHN Daily Trial, who, by virtue of their willingness to be randomized into a trial potentially requiring a more burdensome and time-consuming therapy, were likely different from the general hemodialysis population. Finally, despite several strengths, bioimpedance still requires a degree of technical and interpretative expertise and remains an imperfect biomarker of true volume status, necessitating some caution in the extrapolation of these results to contemporary clinical practice.

In conclusion, among patients in the FHN Daily Trial, we observed that decreases in vector length from baseline to 12 months were associated with increases in cardiac MRI parameters of LV mass and volume over the same period. These findings did not differ according to the randomized treatment arm, suggesting that improved volume control may be a potential mechanism for improvements in cardiac structure and function.

## Supplementary Material

## Data Availability

Anonymized data created for the study are or will be available in a persistent repository upon publication. Analyzable Data; Clinical Trial Data; Published Material. NIDDK Repository. https://repository.niddk.nih.gov/studies/fhn_daily/.

## Supplemental Material

Supplemental Table 1. Baseline characteristics of included and excluded participants.

Supplemental Table 2. Association of the change in vector length with the changes in cardiac MRI index parameters (models including adjustment for sodium gradient).

Supplemental Table 3. Association of the change in vector length with the changes in cardiac MRI index parameters according to subgroups of randomized treatment arm (3/wk or 6/wk hemodialysis).

Supplemental Figure 1. Restricted cubic splines showing the adjusted association of mean changes in vector length with the mean changes in cardiac MRI parameters.

## References

[1] Johansen KL Chertow GM Foley RN, . US renal data system 2020 annual data report: epidemiology of kidney disease in the United States. Am J Kidney Dis. 2021;77(4 suppl 1):A7–A8. doi:10.1053/j.ajkd.2021.01.002 33752804 PMC8148988

[2] Foley RN Parfrey PS Harnett JD, . Clinical and echocardiographic disease in patients starting end-stage renal disease therapy. Kidney Int. 1995;47(1):186–192. doi:10.1038/ki.1995.22 7731145

[3] London GM Pannier B Guerin AP, . Alterations of left ventricular hypertrophy in and survival of patients receiving hemodialysis: follow-up of an interventional study. J Am Soc Nephrol. 2001;12(12):2759–2767. doi:10.1681/ASN.V12122759 11729246

[4] McCullough PA Chan CT Weinhandl ED Burkart JM Bakris GL. Intensive hemodialysis, left ventricular hypertrophy, and cardiovascular disease. Am J Kidney Dis. 2016;68(5S1):S5–S14. doi:10.1053/j.ajkd.2016.05.025 27772643

[5] Silberberg JS Barre PE Prichard SS Sniderman AD. Impact of left ventricular hypertrophy on survival in end-stage renal disease. Kidney Int. 1989;36(2):286–290. doi:10.1038/ki.1989.192 2528654

[6] Chan CT Floras JS Miller JA Richardson RMA Pierratos A. Regression of left ventricular hypertrophy after conversion to nocturnal hemodialysis. Kidney Int. 2002;61(6):2235–2239. doi:10.1046/j.1523-1755.2002.00362.x 12028465

[7] White HD Norris RM Brown MA Brandt PW Whitlock RM Wild CJ. Left ventricular end-systolic volume as the major determinant of survival after recovery from myocardial infarction. Circulation. 1987;76(1):44–51. doi:10.1161/01.cir.76.1.44 3594774

[8] De Winter O De Sutter J Dierckx RA. Clinical relevance of left ventricular volume assessment by gated myocardial SPET in patients with coronary artery disease. Eur J Nucl Med Mol Imaging. 2002;29(7):957–966. doi:10.1007/s00259-002-0828-z 12111136

[9] Wang Y Gu Z. Effect of bioimpedance-defined overhydration parameters on mortality and cardiovascular events in patients undergoing dialysis: a systematic review and meta-analysis. J Int Med Res. 2021;49(9):03000605211031063. doi:10.1177/03000605211031063 34496645 PMC8438275

[10] Martin LC Franco RJS Gavras I, . Association between hypervolemia and ventricular hypertrophy in hemodialysis patients. Am J Hypertens. 2004;17(12 Pt 1):1163–1169. doi:10.1016/j.amjhyper.2004.07.017 15607624

[11] Torino C Gargani L Sicari R, . The agreement between auscultation and lung ultrasound in hemodialysis patients: the LUST study. Clin J Am Soc Nephrol. 2016;11(11):2005–2011. doi:10.2215/CJN.03890416 27660305 PMC5108194

[12] Suri RS Garg AX Chertow GM, . Frequent Hemodialysis Network (FHN) randomized trials: study design. Kidney Int. 2007;71(4):349–359. doi:10.1038/sj.ki.5002032 17164834

[13] Sergeyeva O Gorodetskaya I Ramos R, . Challenges to enrollment and randomization of the frequent hemodialysis Network (FHN) daily trial. J Nephrol. 2012;25(3):302–309. doi:10.5301/jn.5000160 22505248

[14] FHN Trial Group. In-center hemodialysis six times per week versus three times per week. N Engl J Med. 2010;363(24):2287–2300. doi:10.1056/NEJMoa1001593 21091062 PMC3042140

[15] Chan CT Greene T Chertow GM, . Determinants of left ventricular mass in patients on hemodialysis: frequent Hemodialysis Network (FHN) Trials. Circ Cardiovasc Imaging. 2012;5(2):251–261. doi:10.1161/CIRCIMAGING.111.969923 22360996 PMC3328963

[16] Ferrario M Raimann JG Larive B, . Non-linear heart rate variability indices in the frequent hemodialysis Network trials of chronic hemodialysis patients. Blood Purif. 2015;40(1):99–108. doi:10.1159/000381665 26159747 PMC4540641

[17] Chan CT Kaysen GA Beck GJ, . The effect of frequent hemodialysis on matrix metalloproteinases, their tissue inhibitors, and FGF23: implications for blood pressure and left ventricular mass modification in the Frequent Hemodialysis Network trials. Hemodial Int. 2020;24(2):162–174. doi:10.1111/hdi.12807 31826326

[18] Kaysen GA Greene T Larive B, . The effect of frequent hemodialysis on nutrition and body composition: frequent hemodialysis Network trial. Kidney Int. 2012;82(1):90–99. doi:10.1038/ki.2012.75 22456602 PMC3328304

[19] Lukaski HC. Biological indexes considered in the derivation of the bioelectrical impedance analysis. Am J Clin Nutr. 1996;64(3 suppl l):397S–404S. doi:10.1093/ajcn/64.3.397S 8780355

[20] Du Bois D Du Bois EF. A formula to estimate the approximate surface area if height and weight be known. 1916. Nutrition (Burbank, Los Angeles County, Calif.) 1989;5(5):303–313; discussion 312-313. PMID: 2520314 2520314

[21] Patel RK Oliver S Mark PB, . Determinants of left ventricular mass and hypertrophy in hemodialysis patients assessed by cardiac magnetic resonance imaging. Clin J Am Soc Nephrol. 2009;4(9):1477–1483. doi:10.2215/CJN.03350509 19713289 PMC2736694

[22] Stewart GA Foster J Cowan M, . Echocardiography overestimates left ventricular mass in hemodialysis patients relative to magnetic resonance imaging. Kidney Int. 1999;56(6):2248–2253. doi:10.1046/j.1523-1755.1999.00786.x 10594802

[23] Zoccali C Benedetto FA Mallamaci F, . Left ventricular mass monitoring in the follow-up of dialysis patients: prognostic value of left ventricular hypertrophy progression. Kidney Int. 2004;65(4):1492–1498. doi:10.1111/j.1523-1755.2004.00530.x 15086493

[24] Zoccali C Benedetto FA Mallamaci F, . Prognostic value of echocardiographic indicators of left ventricular systolic function in asymptomatic dialysis patients. J Am Soc Nephrol. 2004;15(4):1029–1037. doi:10.1097/01.ASN.0000117977.14912.91 15034106

[25] Koc Y Unsal A Kayabasi H, . Impact of volume status on blood pressure and left ventricle structure in patients undergoing chronic hemodialysis. Ren Fail. 2011;33(4):377–381. doi:10.3109/0886022X.2011.565139 21529265

[26] Agarwal R. Hypervolemia is associated with increased mortality among hemodialysis patients. Hypertension. 2010;56(3):512–517. doi:10.1161/HYPERTENSIONAHA.110.154815 20625076 PMC2929660

[27] Fagugli RM Pasini P Quintaliani G, . Association between extracellular water, left ventricular mass and hypertension in haemodialysis patients. Nephrol Dial Transplant. 2003;18(11):2332–2338. doi:10.1093/ndt/gfg371 14551362

[28] Han BG Lee JY Choi SO Yang JW Kim JS. Relative overhydration is independently associated with left ventricular hypertrophy in dialysis naïve patients with stage 5 chronic kidney disease. Sci Rep. 2020;10(1):15924. doi:10.1038/s41598-020-73038-8 33009458 PMC7532187

[29] Han BG Lee JY Kim MR, . Fluid overload is a determinant for cardiac structural and functional impairments in type 2 diabetes mellitus and chronic kidney disease stage 5 not undergoing dialysis. PLoS One. 2020;15(7):e0235640. doi:10.1371/journal.pone.0235640 32730268 PMC7392282

[30] Chan CT Greene T Chertow GM, . Effects of frequent hemodialysis on ventricular volumes and left ventricular remodeling. Clin J Am Soc Nephrol. 2013;8(12):2106–2116. doi:10.2215/CJN.03280313 23970131 PMC3848394

[31] Fagugli RM Reboldi G Quintaliani G, . Short daily hemodialysis: blood pressure control and left ventricular mass reduction in hypertensive hemodialysis patients. Am J Kidney Dis. 2001;38(2):371–376. doi:10.1053/ajkd.2001.26103 11479164

[32] Flythe JE Mc Causland FR. Dialysate sodium: rationale for evolution over time. Semin Dial. 2017;30(2):99–111. doi:10.1111/sdi.12570 28066913 PMC5334180

[33] Marshall MR Vandal AC de Zoysa JR, . Effect of low-sodium versus conventional sodium dialysate on left ventricular mass in home and self-care satellite facility hemodialysis patients: a randomized clinical trial. J Am Soc Nephrol. 2020;31(5):1078–1091. doi:10.1681/ASN.2019090877 32188697 PMC7217404

