# Supplementary material for: Association of Changes in Vector Length with Changes in Left Ventricular Mass among Patients on Maintenance Hemodialysis: A Secondary Analysis of the Frequent Hemodialysis Network Daily Trial
Source: Kidney360. 2024 Apr 24;5(6):870–6. doi: 10.34067/KID.0000000000000443 (PMC11219120; doi:10.34067/KID.0000000000000443)
Supplement: Supplementary file 1 [file kidney360-5-870-s001.pdf]

ASN American Society of Nephrology JASN Journal of the American Society of Nephrology KIDNEY360 Accessing Our World From Every Angle CJASN Clinical Journal of the American Society of Nephrology

ASN Journal Disclosure Form

As per ASN journal policy, I have disclosed any financial relationship or commitment held by myself and/or my spouse/partner in the past 36 months as included below. I have listed my Current Employer below to indicate there is a relationship requiring disclosure. If no relationship exists, my Current Employer is not listed.

G. Chertow reports the following:
Employer: Stanford University School of Medicine; Consultancy: Akebia, Ardelyx, AstraZeneca, Calico, Gilead, Miromatrix, Reata, Sanifit, Unicycive, Vertex; Ownership Interest: Ardelyx, CloudCath, Durect, DxNow, Eliaz Therapeutics, Outset, Renibus, Unicycive; Research Funding: NIDDK, NIAID, CSL Behring; Advisory or Leadership Role: Board of Directors, Satellite Healthcare, Co-Editor, Brenner & Rector's The Kidney (Elsevier); and Other Interests or Relationships: DSMB service: NIDDK, Bayer, Gilead, Mineralys, ReCor.

I understand that the information above will be published within the journal article, if accepted, and that failure to comply and/or to accurately and completely report the potential financial conflicts of interest could lead to the following: 1) Prior to publication, article rejection, or 2) Post-publication, sanctions ranging from, but not limited to, issuing a correction, reporting the inaccurate information to the authors' institution, banning authors from submitting work to ASN journals for varying lengths of time, and/or retraction of the published work.

Name: Glenn Chertow
Manuscript ID: K360-2024-000142R1
Manuscript Title: Association of changes in vector length with changes in left ventricular mass among patients on maintenance hemodialysis
Date of Completion: March 6, 2024
Disclosure Updated Date: October 4, 2023

ASN American Society of Nephrology | JASN Journal of the American Society of Nephrology | KIDNEY360 Accessing Our World From Every Angle | CJASN Clinical Journal of the American Society of Nephrology

ASN Journal Disclosure Form

As per ASN journal policy, I have disclosed any financial relationship or commitment held by myself and/or my spouse/partner in the past 36 months as included below. I have listed my Current Employer below to indicate there is a relationship requiring disclosure. If no relationship exists, my Current Employer is not listed.

Y. Farag reports the following:
Employer: Alexion AstraZeneca Rare Disease Unit

I understand that the information above will be published within the journal article, if accepted, and that failure to comply and/or to accurately and completely report the potential financial conflicts of interest could lead to the following: 1) Prior to publication, article rejection, or 2) Post-publication, sanctions ranging from, but not limited to, issuing a correction, reporting the inaccurate information to the authors' institution, banning authors from submitting work to ASN journals for varying lengths of time, and/or retraction of the published work.

Name: Youssef MK Farag
Manuscript ID: K360-2024-000142R1
Manuscript Title: Association of changes in vector length with changes in left ventricular mass among patients on maintenance hemodialysis
Date of Completion: March 7, 2024
Disclosure Updated Date: March 7, 2024

ASN American Society of Nephrology | JASN Journal of the American Society of Nephrology | KIDNEY360 Accessing Our World From Every Angle | CJASN Clinical Journal of the American Society of Nephrology

ASN Journal Disclosure Form

As per ASN journal policy, I have disclosed any financial relationship or commitment held by myself and/or my spouse/partner in the past 36 months as included below. I have listed my Current Employer below to indicate there is a relationship requiring disclosure. If no relationship exists, my Current Employer is not listed.

F. McCausland reports the following:
Employer: Brigham and Women's Hospital; Consultancy: GlaxoSmithKline; Zydus Therapeutics Inc.; Research Funding: Research Funding paid to institution from NIDDK, Satellite Healthcare, Novartis, Lexicon, and Fifth Eye.; and Other Interests or Relationships: Expert witness fees from Rubin-Anders scientific.

I understand that the information above will be published within the journal article, if accepted, and that failure to comply and/or to accurately and completely report the potential financial conflicts of interest could lead to the following: 1) Prior to publication, article rejection, or 2) Post-publication, sanctions ranging from, but not limited to, issuing a correction, reporting the inaccurate information to the authors' institution, banning authors from submitting work to ASN journals for varying lengths of time, and/or retraction of the published work.

Name: Finnian R. McCausland
Manuscript ID: K360-2024-000142R1
Manuscript Title: Association of changes in vector length with changes in left ventricular mass among patients on maintenance hemodialysis
Date of Completion: March 6, 2024
Disclosure Updated Date: March 6, 2024

ASN American Society of Nephrology | JASN Journal of the American Society of Nephrology | KIDNEY360 Accessing Our World From Every Angle | CJASN Clinical Journal of the American Society of Nephrology

ASN Journal Disclosure Form

As per ASN journal policy, I have disclosed any financial relationship or commitment held by myself and/or my spouse/partner in the past 36 months as included below. I have listed my Current Employer below to indicate there is a relationship requiring disclosure. If no relationship exists, my Current Employer is not listed.

K. Ravi reports the following:
Employer: Brigham and Women's Hospital; and Ownership Interest: Halo LLC (spouse).

I understand that the information above will be published within the journal article, if accepted, and that failure to comply and/or to accurately and completely report the potential financial conflicts of interest could lead to the following: 1) Prior to publication, article rejection, or 2) Post-publication, sanctions ranging from, but not limited to, issuing a correction, reporting the inaccurate information to the authors' institution, banning authors from submitting work to ASN journals for varying lengths of time, and/or retraction of the published work.

Name: Katherine Scovner Ravi
Manuscript ID: K360-2024-000142R1
Manuscript Title: Association of changes in vector length with changes in left ventricular mass among patients on maintenance hemodialysis
Date of Completion: March 6, 2024
Disclosure Updated Date: March 6, 2024

ASN
American Society of Nephrology
JASN
Journal of the American Society of Nephrology
KIDNEY360
Accessing Our World From Every Angle
CJASN
Clinical Journal of the American Society of Nephrology

ASN Journal Disclosure Form

As per ASN journal policy, I have disclosed any financial relationship or commitment held by myself and/or my spouse/partner in the past 36 months as included below. I have listed my Current Employer below to indicate there is a relationship requiring disclosure. If no relationship exists, my Current Employer is not listed.

E. Sayed has nothing to disclose.

I understand that the information above will be published within the journal article, if accepted, and that failure to comply and/or to accurately and completely report the potential financial conflicts of interest could lead to the following: 1) Prior to publication, article rejection, or 2) Post-publication, sanctions ranging from, but not limited to, issuing a correction, reporting the inaccurate information to the authors' institution, banning authors from submitting work to ASN journals for varying lengths of time, and/or retraction of the published work.

Name: Enass Sayed
Manuscript ID: (K360-2024-000142R1)
Manuscript Title: ("Association of changes in vector length with changes in left ventricular mass among patients on maintenance hemodialysis")
Date of Completion: March 7, 2024
Disclosure Updated Date: March 7, 2024
