# Supplementary material for: Association of Changes in Vector Length with Changes in Left Ventricular Mass among Patients on Maintenance Hemodialysis: A Secondary Analysis of the Frequent Hemodialysis Network Daily Trial
Source: Kidney360. 2024 Apr 24;5(6):870–6. doi: 10.34067/KID.0000000000000443 (PMC11219120; doi:10.34067/KID.0000000000000443)
Supplement: Supplementary file 2 [file kidney360-5-870-s002.pdf]

Supplementary Material

Supplementary Table S1. Baseline characteristics of included and excluded participants

Supplementary Table 2. Association of the change in vector length with the changes in cardiac MRI indices parameters (models including adjustment for sodium gradient).

Supplementary Table 3. Association of the change in vector length with the changes in cardiac MRI indices parameters according to sub-groups of randomized treatment arm (3/week or 6/week hemodialysis).

Supplementary Figure 1. Restricted cubic splines showing the adjusted association of mean changes in vector length with the mean changes in cardiac MRI parameters.

Supplementary Table S1. Baseline characteristics of included and excluded participants

Characteristic	Included participants<br>n=160	Excluded participants<br>n=85	P-value
**Age, yrs**	50 ±13	51 ±15	0.52
**Female, n(%)**	56 (35.0%)	38 (44.7%)	0.14
**Race or Ethnic group, n (%)**			0.18
- **Native American, Aboriginal Canadian, Alaskan Native, or First Nation**	6 (3.8%)	2 (2.4%)	
- **Asian**	13 (8.1%)	3 (3.5%)	
- **Native Hawaiian or another Pacific Islander**	3 (1.9%)	1 (1.2%)	
- **Black**	72 (45.0%)	30 (35.3%)	
- **White**	49 (30.6%)	40 (47.1%)	
- **Multiracial, unknown or not reported**	17 (10.6%)	9 (10.6%)	
**BMI, kg/m^2**	27.2 ± 6.6	28.3± 7.0	0.19
**Dialysis Access, n (%)**			0.69
- **Graft**	26 (16.5%)	17 (21.0%)	
- **Fistula**	101 (63.9%)	49 (60.5%)	
- **Catheter**	31 (19.6%)	15 (18.5%)	
**Duration of ESKD, n (%)**			

- **< 2 years**	47 (29.4%)	18 (21.2%)	0.37
- **2-5 years**	50 (31.3%)	31 (36.5%)	
- **> 5 years**	63 (39.4%)	36 (42.4%)	
**Coexisting Medical Conditions, n (%)**			
- **Hypertension**	147 (91.9%)	72 (84.7%)	0.08
- **Heart Failure**	31 (19.4%)	18 (21.2%)	0.74
- **Diabetes Mellitus**	62 (38.8%)	38 (44.7%)	0.37
**KrU, n (%)**			0.57
- **Anuria**	104 (65.0%)	58 (68.2%)	
- **> 0- 1 ml/min**	23 (14.4%)	14 (16.5%)	
- **> 1- 3 ml/min**	31 (19.4%)	11 (12.9%)	
- **> 3 ml/min**	2 (1.3%)	2 (2.4%)	
**Pre-dialysis Systolic BP, mmHg**	148 ± 18	145 ± 17	0.22
**Pre-dialysis laboratory measurements**			
- **Hemoglobin, g/dL**	12.0 ±1.3	11.8 ± 1.3	0.10
- **Phosphorus, mg/dL**	5.8 ± 1.6	5.7 ± 1.7	0.76
**Equilibrated Kt/V urea**	1.4 ± 0.3	1.4 ± 0.3	0.69
**Ultrafiltration rate, mL/kg/hour**	12 ± 4	11 ± 4	0.07
**Sodium gradient, mmol/L**	-2 [-4, 1]	-1 [-4, 1]	0.52
**ACEi or ARB use, n(%)**	78 (48.8%)	40 (47.1%)	0.80

**Erythropoietin dose, Units**	8188 [3150, 17125]	11250 [5625, 19800]	0.08
**Randomized to 6/week HD, n(%)**	86 (53.8%)	39 (45.9%)	0.24

*Results are presented as mean ± standard deviation, or median [25th-75th percentiles] for continuous variables.
Abbreviations: BMI, body mass index; ESKD, end-stage kidney disease; KrU, residual renal urea clearance; BP, blood pressure; HD, hemodialysis; ACEi, angiotensin-converting enzyme inhibitor; ARB, angiotensin receptor blocker

Supplementary Table 2. Association of the change in vector length with the changes in cardiac MRI indices parameters (models including adjustment for sodium gradient).

		Change in outcome from baseline to month 12 according to change in vector length (95%CI)					
**Outcome**	Model	Per 50 Ω/m decrease in vector length	P- value	Tertile 1	Tertile 2	Tertile 3	P-trend
**LVM, g**	Unadjusted	10.1 (4.6, 15.6)	<0.001	15.0 (2.7, 27.3)	9.3 (-3.1, 21.6)	Ref	0.02
	Adjusted	13.5 (4.2, 22.7)	0.01	17.6 (-1.5, 36.8)	10.3 (-5.9, 26.5)	Ref	0.06
**LVMI, g/m^2**	Unadjusted	5.1 (2.1, 8.0)	0.001	6.7 (0.2, 13.3)	4.3 (-2.3, 10.9)	Ref	0.04
	Adjusted	5.5 (0.6, 10.5)	0.03	5.9 (-4.3, 16.0)	4.2 (-4.4, 12.9)	Ref	0.22
**LVEDV, mL**	Unadjusted	16.3 (9.2, 23.4)	<0.001	27.5 (11.6, 43.4)	23.3 (7.4, 39.3)	Ref	0.001
	Adjusted	20.4 (11.2, 29.7)	<0.001	31.3 (11.7, 50.8)	15.4 (-1.2, 31.9)	Ref	0.002
**LVESV, mL**	Unadjusted	9.4 (4.7, 14.0)	<0.001	13.4 (2.9, 23.9)	15.8 (5.3, 26.3)	Ref	0.01
	Adjusted	10.6 (3.5, 17.8)	0.004	13.2 (-1.7, 28.0)	9.7 (-2.9, 22.2)	Ref	0.06
**LVSV, mL**	Unadjusted	6.9 (2.2, 11.6)	0.004	14.1 (3.7, 24.4)	7.5 (-2.9, 17.9)	Ref	0.01
	Adjusted	9.8 (3.4, 16.2)	0.003	16.9 (4.0, 29.9)	5.5 (-5.5, 16.5)	Ref	0.01
**LVEF, %**	Unadjusted	-1.7 (-3.4, 0.1)	0.06	-1.8 (-5.6, 2.0)	-3.1 (-6.9, 0.7)	Ref	0.36
	Adjusted	-1.1 (-4.1, 1.9)	0.48	-0.7 (-6.7, 5.4)	-1.3 (-6.4, 3.7)	Ref	0.77

The multivariable model adjusted for baseline vector length, baseline outcome, randomized treatment assignment, age, sex, race, body mass index, access type, vintage (<2, 2-5, >5 years), pre-dialysis systolic BP, hypertension, heart failure, diabetes, residual urea clearance (0, ≤1, >1 to 3, >3 ml/min), hemoglobin, phosphate, ultrafiltration rate, angiotensin-converting enzyme inhibitor (ACEi) or angiotensin receptor blocker (ARB) use, log-transformed erythropoietin dose, equilibrated Kt/V, and sodium gradient.

Abbreviations: LVMI, left ventricular mass index; LVM, left ventricular mass; LVEDV, left ventricular end-diastolic volume; LVESV, left ventricular end-systolic volume; LVSV, left ventricular stroke volume; LVEF, left ventricular ejection fraction.

Supplementary Table 3. Association of the change in vector length with the changes in cardiac MRI indices parameters according to sub-groups of randomized treatment arm (3/week or 6/week hemodialysis).

		Change in outcome from baseline to month 12 according to change in vector length (95%CI)					
**Outcome**	**Model**	**Per 50 Ω/m decrease in vector length**	**P-value**	**Tertile 1**	**Tertile 2**	**Tertile 3**	**P-trend**
**Randomized to 3/week HD**							
**LVM, g**	Unadjusted	7.6 (0.3, 14.9)	0.04	11.0 (-5.1, 27.2)	2.4 (-14.3, 19.2)	Ref	0.13
	Adjusted	11.3 (-2.6, 25.3)	0.11	13.8 (-13.9, 41.6)	4.3 (-22.6, 31.2)	Ref	0.30
**LVMI, g/m^2**	Unadjusted	3.4 (-0.6, 7.5)	0.10	4.6 (-4.4, 13.5)	0.4 (-8.9, 9.7)	Ref	0.24
	Adjusted	4.9 (-2.8, 12.5)	0.21	5.5 (-9.5, 20.6)	2.0 (-12.7, 16.6)	Ref	0.44
**LVEDV, mL**	Unadjusted	16.0 (5.1, 26.9)	0.01	25.5 (1.2, 49.8)	7.3 (-18.0, 32.6)	Ref	0.03
	Adjusted	24.9 (9.3, 40.5)	0.003	35.9 (4.0, 67.7)	18.2 (-12.8, 49.2)	Ref	0.03
**LVESV, mL**	Unadjusted	8.8 (1.2, 16.4)	0.02	11.3 (-5.7, 28.3)	12.1 (-5.6, 29.8)	Ref	0.26
	Adjusted	10.6 (-1.2, 22.4)	0.08	15.4 (-7.5, 38.3)	17.3 (-4.9, 39.6)	Ref	0.21
**LVSV, mL**	Unadjusted	7.2 (-0.3, 14.8)	0.06	14.2 (-1.8, 30.3)	-4.8 (-21.5, 11.9)	Ref	0.03
	Adjusted	14.3 (2.6, 26.1)	0.02	20.0 (-3.3, 43.2)	1.6 (-21.2, 24.4)	Ref	0.07
**LVEF, %**	Unadjusted	-1.3 (-4.4, 1.7)	0.39	-1.4 (-8.0, 5.2)	-6.0 (-12.9, 0.8)	Ref	0.97
	Adjusted	0.4 (-4.4, 5.2)	0.87	-0.6 (-9.7, 8.5)	-4.5 (-13.5, 4.5)	Ref	0.98

**Randomized to 6/week HD**							
**LVM, g**	Unadjusted	9.7 (1.5, 18.0)	0.02	9.9 (-9.8, 29.7)	10.5 (-7.8, 28.7)	Ref	0.26
	Adjusted	-1.3 (-10.3, 7.7)	0.77	-8.5 (-28.2, 11.1)	-3.5 (-22.6, 15.6)	Ref	0.39
**LVMI, g/m²**	Unadjusted	5.2 (0.9, 9.5)	0.02	4.5 (-5.9, 14.8)	5.3 (-4.3, 14.9)	Ref	0.33
	Adjusted	-1.4 (-6.5, 3.7)	0.58	-8.1 (-19.1, 2.8)	-2.0 (-12.9, 8.9)	Ref	0.15
**LVEDV, mL**	Unadjusted	13.5 (3.7, 23.2)	0.01	14.3 (-8.5, 37.0)	30.9 (9.9, 52.0)	Ref	0.11
	Adjusted	7.5 (-2.0, 17.0)	0.12	13.9 (-7.7, 35.4)	12.4 (-7.8, 32.7)	Ref	0.17
**LVESV, mL**	Unadjusted	8.9 (2.5, 15.2)	0.01	11.2 (-3.8, 26.2)	16.8 (2.9, 30.7)	Ref	0.08
	Adjusted	2.8 (-4.0, 9.6)	0.41	2.9 (-12.4, 18.2)	3.1 (-11.3, 17.5)	Ref	0.67
**LVSV, mL**	Unadjusted	4.6 (-1.6, 10.8)	0.14	3.1 (-11.2, 17.3)	14.1 (0.9, 27.4)	Ref	0.45
	Adjusted	4.6 (-1.3, 10.5)	0.13	12.1 (-0.9, 25.2)	8.3 (-4.0, 20.7)	Ref	0.06
**LVEF, %**	Unadjusted	-1.9 (-4.1, 0.2)	0.08	-3.3 (-8.4, 1.7)	-0.8 (-5.4, 3.9)	Ref	0.21
	Adjusted	-0.5 (-3.2, 2.3)	0.73	0.4 (-5.6, 6.5)	1.8 (-3.9, 7.5)	Ref	0.84

The multivariable model adjusted for baseline vector length, baseline outcome, randomized treatment assignment, age, sex, race, body mass index, access type, vintage (<2, 2-5, >5 years), pre-dialysis systolic BP, hypertension, heart failure, diabetes, residual urea clearance (0, ≤1, >1 to 3, >3 ml/min), hemoglobin, phosphate, ultrafiltration rate, angiotensin-converting enzyme inhibitor (ACEi) or angiotensin receptor blocker (ARB) use, log-transformed erythropoietin dose, and equilibrated Kt/V.

Abbreviations: LVMI, left ventricular mass index; LVM, left ventricular mass; LVEDV, left ventricular end-diastolic volume; LVESV, left ventricular end-systolic volume; LVSV, left ventricular stroke volume; LVEF, left ventricular ejection fraction.

Supplementary Figure 1. Restricted cubic splines showing the adjusted association of mean changes in vector length with the mean changes in cardiac MRI parameters.

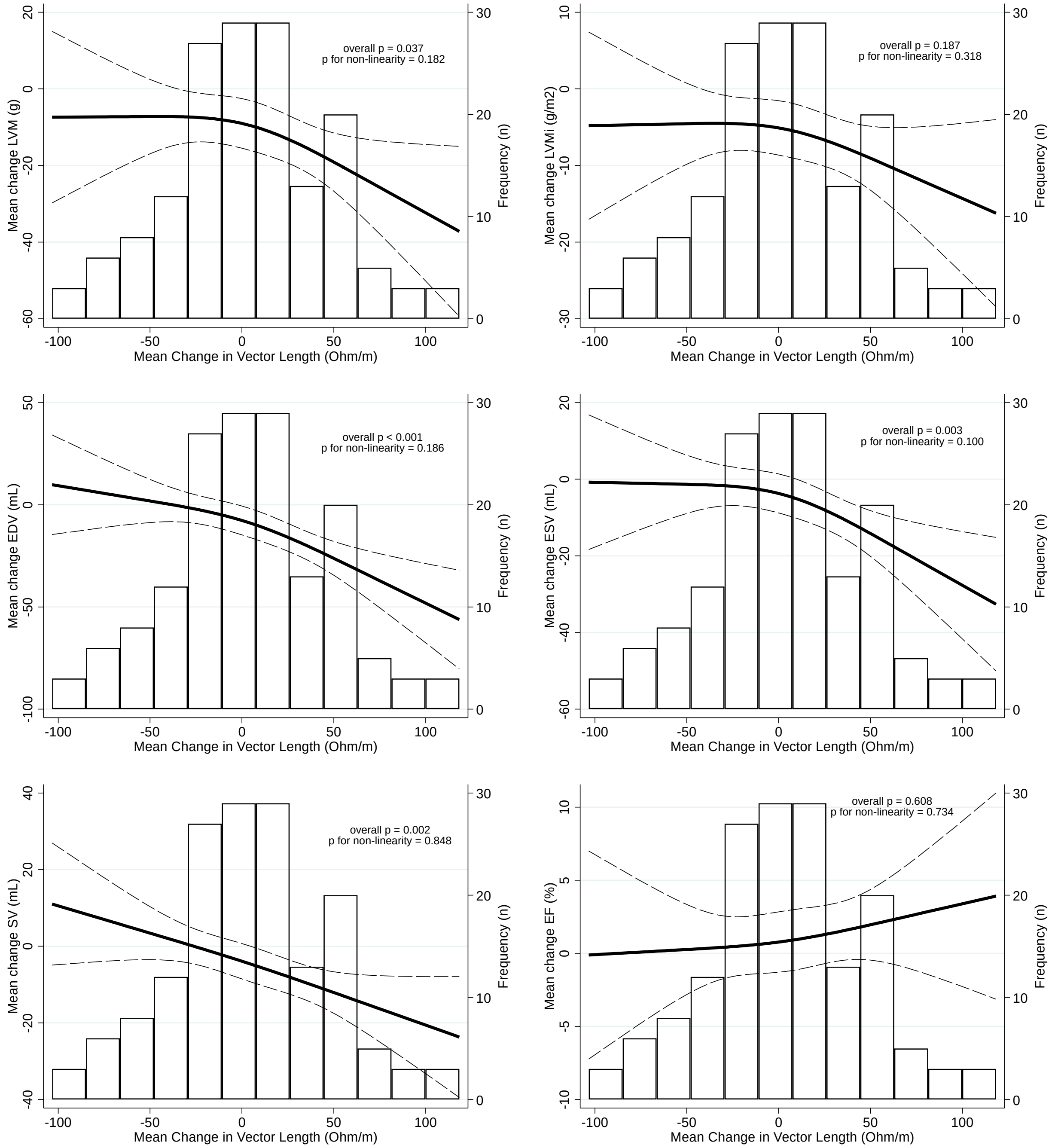

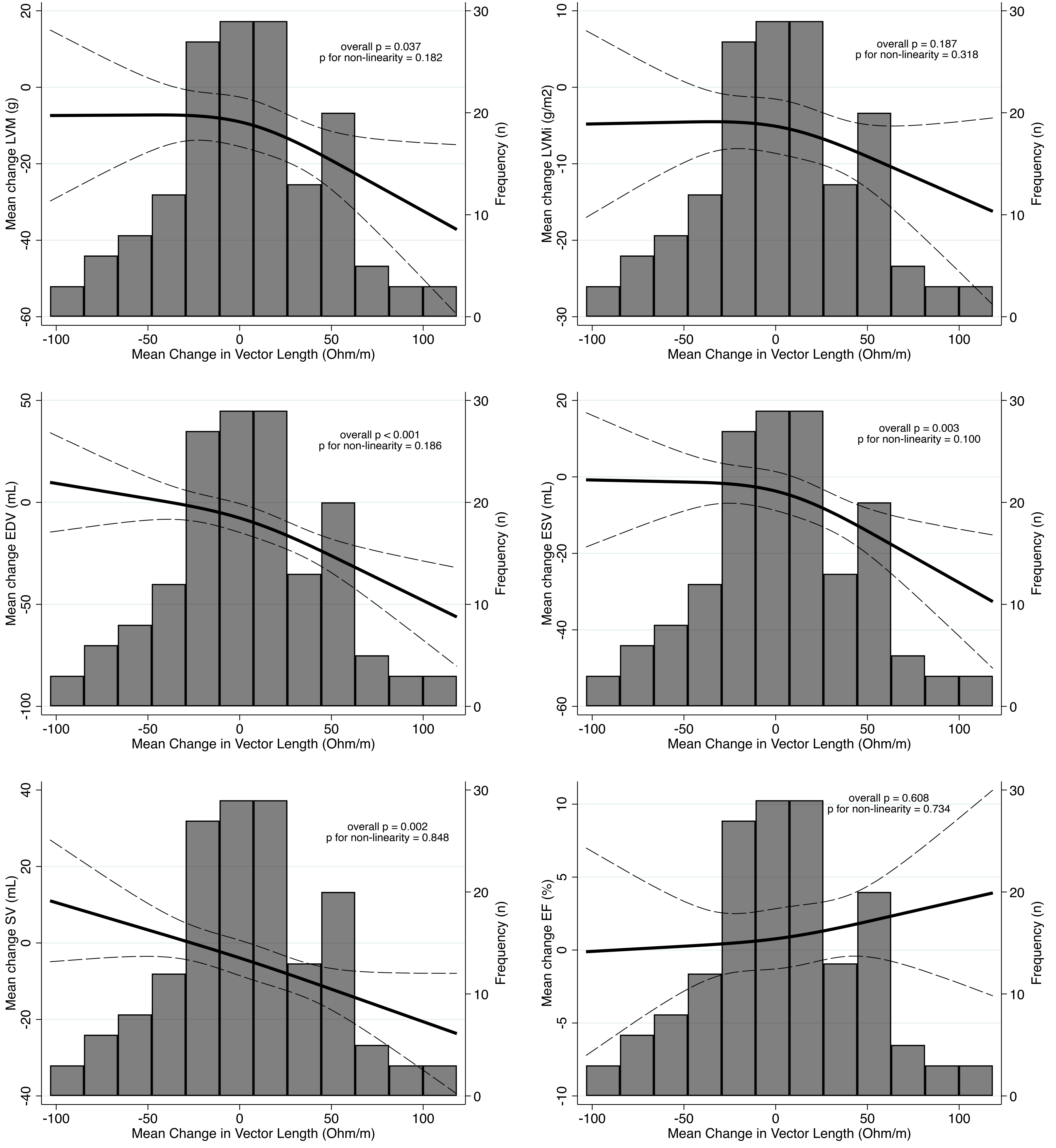

overall p = 0.037
p for non-linearity = 0.182
Mean change LVM (g)
Frequency (n)
Mean Change in Vector Length (Ohm/m)
overall p = 0.187
p for non-linearity = 0.318
Mean change LVMi (g/m2)
Frequency (n)
Mean Change in Vector Length (Ohm/m)
overall p < 0.001
p for non-linearity = 0.186
Mean change EDV (mL)
Frequency (n)
Mean Change in Vector Length (Ohm/m)
overall p = 0.003
p for non-linearity = 0.100
Mean change ESV (mL)
Frequency (n)
Mean Change in Vector Length (Ohm/m)
overall p = 0.002
p for non-linearity = 0.848
Mean change SV (mL)
Frequency (n)
Mean Change in Vector Length (Ohm/m)
overall p = 0.608
p for non-linearity = 0.734
Mean change EF (%)
Frequency (n)
Mean Change in Vector Length (Ohm/m)
